# Temporal variations of ecological security with soil and water loss stress in black soil region of northeast China: a case study on Baiquan County

**DOI:** 10.1186/2193-1801-2-S1-S6

**Published:** 2013-12-11

**Authors:** Liying Sun, Zhenju Liu, Mingguo Zheng, Qiangguo Cai, Haiyan Fang

**Affiliations:** Key Laboratory of Water Cycle and Related Surface Processes, Institute of Geographical Sciences and Resources Research, Chinese Academy of Sciences, Beijing, 100101 China; Guangxi Hydraulic Research Institute, Nanning, 530023 China

**Keywords:** temporal variation, black soil region, ecological security, soil and water loss

## Abstract

The deterioration of ecological situation with serious soil and water loss in black soil region of northeast China has attracted more attention due to its significant role on food security of China. To investigate the temporal characteristics of ecological status in typical black soil areas, Baiquan County is selected. Based on the model of Press-Status-Response (P-S-R), indicators are established and the ecological security situations with soil and water loss of Baiquan County are evaluated for the years of 1979, 1990, 2000 and 2005. The results show that the ecological insecurity indicator changes from 0.701 to 0.435 from 1979 to 2005, with a decrease of 37.9% for Baiquan County. And the contributions of physical and human factors to the temporal variations of the ecological security are discussed in detail. Moreover, several problems are recognized to be the potential threats to the ecological security in Baiquan county, including reduction of the effective thickness, excessive application of the fertilizer and low efficiency of the agricultural irrigation system. It is found that effective soil and water loss control actions have made great contribution to the improvement of the ecological security in Baiquan county. All these results and discussions are very helpful for the further investigation on the quantitative relationship between soil and water loss and ecological security in black soil region of northeast China.

## Introduction

The concept of ecological security has been widely considered after it was proposed by the government of the United States [1]. Generally, the broad-sense conception of ecological security includes the complex systems of natural, economic and social system, while the narrow-sense conception of ecological security focused on the natural and human influenced ecosystems [2, 3]. As the significant role of the ecological security on the sustainable development, how to establish scientific evaluation methods to measure different levels of the ecological security is key aspect of the related studies [4]. However, there is no agreement on the universally accepted definition, nor the standard assessment approaches [3].

Different assessment approaches and models, such as ecological foot print method, Press-Status-Response (P-S-R) model, cellular automata model and security patterns and surface model, are proposed for various ecosystems and requirements of different time-space dimensions [3, 5–7]. P-S-R framework model could illustrate the causality between the driving pressures and their results, and potential influencing factors could be found through the analysis of the assessment results [8]. Thus, comprehensive evaluation method based on P-S-R framework model is used in this paper.

Black soil region of Northeast China, one of the three typical black soil regions in the world, performs significant roles on the crop production and food security in China [9]. However, serious soil and water loss has caused the degradation of the ecological system due to unreasonable human cultivation and the special physical background of the local region [10]. Efficient soil and water control actions have been taken since 1980s. The major purpose of this paper is to investigate the temporal variations of ecological security with soil and water loss stress in black soil region of Northeast China during the period of 1979 to 2005. Potential problems to the ecological security and effects of soil and water control on the changes of the ecological security were discussed for the further actions to protect the valuable black soil resources.

## Study area and evaluation method

### Study area

Baiquan county is located at 125°30' E~126°31' E and 47°20' N~47°55' N, where is the hinterland of typical black soil zone in Northeast China with a population of approximately 0.55 million inhabitants. The total land area of Baiquan county is about 0.36 million hm^2^. The altitude of Baiquan county is from 240 m to 280 m. Rolling hill regions are taking approximately 77% of the county's total land area, characterized by long slopes of the ramp. The majority of slope lengths in rolling hill regions are from 300 m to 500 m, and it could be up to be more than 1 km.

The total water resources of Baiquan county is 0.47 billion m^3^ and the average annual runoff depth is 45.97 mm with the annual precipitation of 490 mm. Although the precipitation is not high, the special topographic conditions, such as the long slopes could increase the runoff yield and concentration area, and unreasonable human activities have made serious soil erosion in rolling hill regions of the county. Up to now, soil erosion area is about 0.21 million hm^2^, accounting for about 58% of the county's total land area. In which, most soil loss happened in cultivated land, achieving 0.17 million hm^2^ and accounting for about 68% of the total cultivated land area in Baiquan county. On the other side, considerable attention has been focused on soil and water conservation and a series of demonstration projects have been implemented in Baiquan County since 1980s.

### Comprehensive evaluation method

Based on the pressure-state-response (P-S-R) framework model, comprehensive evaluation method is used for the assessment of the ecological security of Baiquan county. Firstly, a four-layer index system is established according to the basic principles, such as representativeness, integrity, stability and availability. Secondly, Weights are determined for each index using the analytic hierarchy process (AHP) method. After that, specific data for four years, 1979, 1990, 2000 and 2005, are collected for the indices in the basic layer of the evaluation index system. Thus the evaluation results could reflect the temporal variations of ecological security with soil and water loss stress in Baiquan county.

#### Evaluation indices

In fact, to establish evaluation indices for the assessment of ecological security is a kind of complicated work, due to the complexity of the ecosystem in different scales and for different concerns. In this paper, the assessment focused on safety of the ecological system with soil and water loss stress in the local scale of Baiquan county. Based on the spirit of the (P-S-R) framework, a four-layer index system with 16 basic indices is established (Table 1). The topmost layer of the index system is a negative index to reflect the ecological security, which means the ecological situation is worsen when the calculated values of this index is higher. Thus it named the 'Ecological Unsafety Index'. Representative factors related to the soil and water loss are selected for the 'pressure', 'status' and 'response' indices, including natural and human factors that influencing the ecological security.

**Table 1:** Evaluation indices for ecological security of Baiquan County

1^st^ layer	2^nd^ layer	3^rd^ layer	4^th^ layer	Weight
Ecological Unsafety Index (*A*)	Pressure *(B*_1_ *)*	Population Pressure (*C*_1_)	Natural Population Growth Rate (*D*_1_)	0.0174
			Population Density (*D*_2_)	0.0174
		Land Pressure (*C*_2_)	Per Capita Arable Land (*D*_3_)	0.0423
			Area Proportion of Soil and water Loss (*D*_4_)	0.1395
		Pollution Pressure (*C*_3_)	Fertilizer Load (*D*_5_)	0.0397
			Pesticide Load (*D*_6_)	0.0397
	Status (*B*_2_)	Environmental Status (*C*_4_)	Probability of Natural Disaster (*D*_7_)	0.0749
			Forestry and Grass Coverage (*D*_8_)	0.0943
			Soil Erosion Modulus (*D*_9_)	0.1188
		Natural Resources Status (*C*_5_)	Effective Thickness of Soil Layer (*D*_10_)	0.1113
			Precipitation Rate of April~May to July~August (*D*_11_)	0.0701
	Response (*B*_3_)	Social Response (*C*_6_)	Effective Irrigation Rate of Arable Land (*D*_12_)	0.0394
		Environmental Response (*C*_7_)	Agricultural Technican Numbers Per Ten Thousand People (*D*_13_)	0.0156
1		Economic Response (*C*_8_)	Area Proportion of Soil and Water Loss Control (*D*_14_)	0.1102
			Per Capita Rural Net Income (*D*_15_)	0.0347
			Agriculture Production Per Unit Cultivated Land (*D*_16_)	0.0347

#### Weight determination

The Analytic Hierarchy Process (AHP) is a structured technique that was developed in the 1970s and has been extensively studied and modified after then [11, 12]. To deal with complex decisions, AHP provides a comprehensive and rational framework. AHP is trying to find a solution that best suits the needs of the decision makers according to their understanding and attitude to the problem. Normally, the AHP includes five steps [12]: (1) decomposing the decision problem into a hierarchy of simpler sub-problems, usually a hierarchy of evaluation indices is established; (2) Building pair-wise comparison matrix in different levels to judge the importance of the elements by paired comparison using the scale method [1, 9]; (3) Determining the relative weight of one element to another through calculating the priority vector and maximum eigenvalue of the comparison matrix; (4) Examination the consistency of the pair-wise comparison matrix in different levels by calculating the consistency index (*CI*) for each level of the hierarchy structures; (5) Calculation the relative weight of the each element to the whole target and examination the consistency of the whole hierarchical matrix. Herein, the process of the weight determining has been programmed using Matlab Soft (Version 7.0). The calculated weights for each index to the 'Ecological Unsafety Index' in the topmost layer of the evaluation indices are listed in Table 1.

#### Data collection and processing

Data for each basic index are collected mainly from the statistical documents published by the local government and related investigations in Baiquan county. The purpose of this article is mainly focused on the temporal variations of ecological security from 1970s to the present. However, the year-to-year data are not available for all indices during this period of time. Thus, according to the availability of the data sources, four years are especially investigated in this article. They are 1979, 1990, 2000 and 2005. As effective soil and water loss control actions were began from 1980s, situations in 1979 are obviously different from those years after 1986. To some extent, this increases the comparability of the investigation results.

Data standardization is necessary to avoid the incomparability of the data for different indices due to their different dimensional units. Single threshold method is used for the data standardization in this article. For positive indices, data standardization is carried out according to formula (1), while it is carried out according to formula (2) for the negative indices. Through the standardization process, the standardized values are in the range of 0[1]. As the topmost index is 'Ecological Unsafety Index'. The increase of the standardized value of each index may suggest the declining of the situation, and the decrease of the standardize value may suggest the improving of the situation.12

In which, the is the standardized value of the initial value (), while is the threshold value of the index.

The determination of threshold value for the basic evaluation index is a key requirement for the data standardization. Local standards and local investigations are used as the reference to determine the threshold value of each basic index of the four-layer evaluation index system to assess ecological security of Baiquan county with soil and water loss stress. The results and their references are listed in Table 2.

**Table 2:** Threshold values for the basic index of the evaluation indices

Basic Index	P/N*	Threshold Value	References
Natural Population Growth Rate (*D*_1_)	P	8 ‰	Wang, 2005 [13]
Population Density (*D*_2_)	P	81.4 person/hm^2^	Average value of Heilongjiang province, 2005
Per Capita Arable Land (*D*_3_)	P	0.43 hm^2^/person	Eco-construction Primary Standard of Heilongjiang Province
Area Proportion of Soil and water Loss (*D*_4_)	P	27.2 %	Target of the starting stage of eco-construction of Heilongjiang province
Fertilizer Load (*D*_5_)	P	200 kg/hm^2^	Construction standards of ecological demonstration areas in Heilongjiang province
Pesticide Load (*D*_6_)	P	4.2 kg/hm^2^	Average value of Heilongjiang province, 2005
Probability of Natural Disaster (*D*_7_)	P	41.5 %	Average value of Heilongjiang province, 2005
Forestry and Grass Coverage (*D*_8_)	N	25 %	Construction standards of ecological demonstration areas in Heilongjiang province
Soil Erosion Modulus (*D*_9_)	P	2500 t/km^2^ a	Soil erosion classification standard
Effective Thickness of Soil Layer (*D*_10_)	P	20 cm	Zhang et al., 2006 [14]
Precipitation Rate of April~May to July~August (*D*_11_)	P	39.3/337.7	Achieved from the analysis of the historical statistical data
Effective Irrigation Rate of Arable Land (*D*_12_)	P	30 mm	Shu K L, 2006 [15]
Agricultural Technican Per Ten Thousand People (*D*_13_)	P	4 person	China rural well-off social standards
Area Proportion of Soil and Water Loss Control (*D*_14_)	N	50 %	Construction standards of ecological demonstration areas in Heilongjiang province
Per Capita Rural Net Income (*D*_15_)	N	2000 RMB	Construction standards of ecological demonstration areas in Heilongjiang province
Agriculture Production Per Unit Cultivated Land (*D*_16_)	N	10000 RMB/hm^2^	Construction standards of circular economy demonstration areas in Heilongjiang province

*p-positive indicator; N-negative indicator.

## Results and discussion

### Assessment results

**Figure 1:**
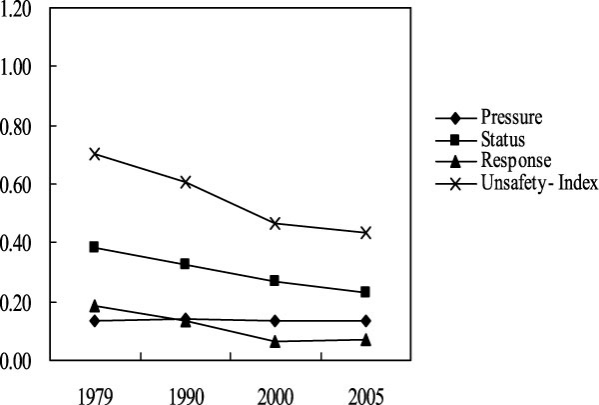
**variations of ecological security from 1979 to 2005 in Baiquan**.

Figure 1 depicts the variations of 'Pressure' (*B*_1_) sub-index, 'Status' (*B*_2_) sub-index, 'Response' (*B*_3_) sub-index and the topmost 'Ecological Unsafety Index' (*A*_1_) of Baiquan county during from 1979 to 2005. The calculated values of *A*_1_ decreased about 37.9%, from 0.701 to 0.435, during the period of 1979~2005, which suggests the ecological situations of Baiquan county are improving during these years.

**Figure 2:**
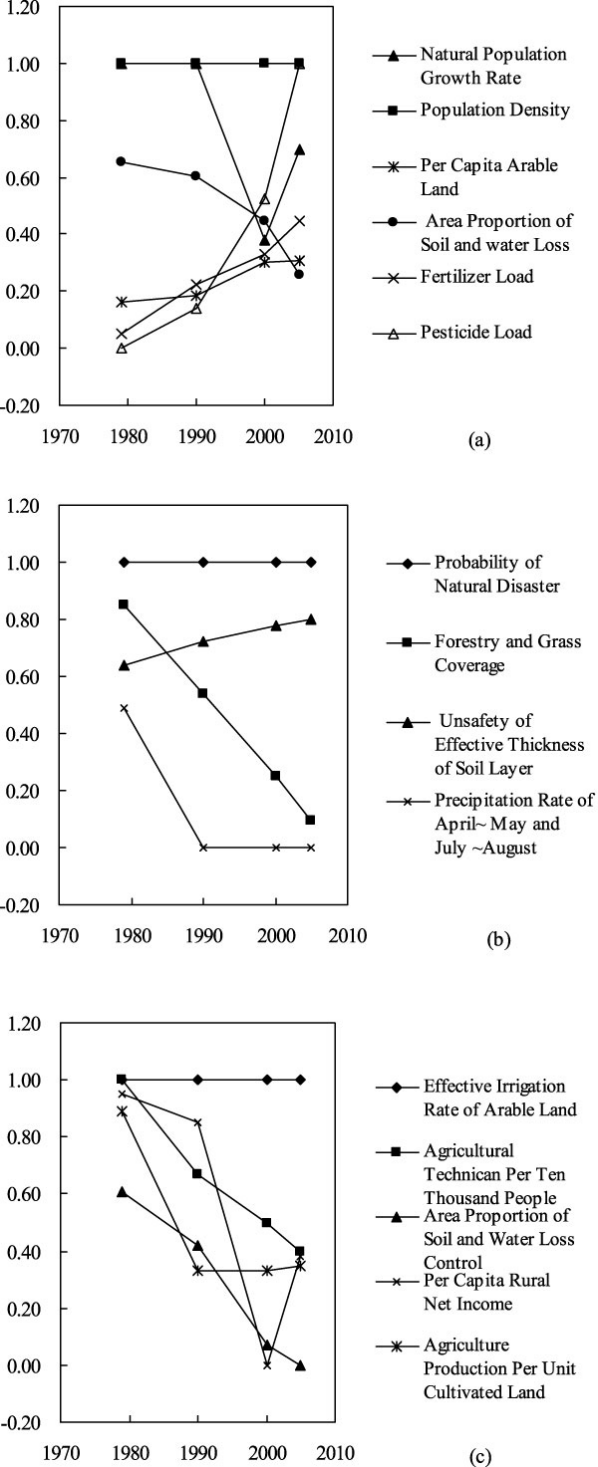
**Temporal variations of sub-indices based on Pressure, Status and Response from 1979 to 2005 in Baiquan County: (a)Temporal variation of sub-indices of 'Pressure ' during 1979~2005; (b) Temporal variation of sub-indices of 'Status ' during 1979~2005; (c) Temporal variation of sub-indices of 'Response' during 1979~2005**.

However, the 'Pressure' sub-index (*B*_1_) does not change a lot during the four years of 1979, 1990, 2000 and 2005. In these four years, the highest value of 'Pressure' sub-index is 0.142 and the lowest value of 'Pressure' value is 0.133. This is mainly due to the mutually counter-balance of the sub-indices of 'Pressure' index. For example, the 'Fertilizer Load' (*D*_5_) and 'Pesticide Load' (*D*_6_) increased greatly since 1979 due to the unreasonable actions of the local farmers (Figure 2a). While, the standardized values of Area Proportion of Soil and water Loss (*D*_4_) decreased a lot due to the demonstration actions of soil and water control in Baiquan County (Figure 2a). And it is noticeable that the four year's standardized values of the Population Density (*D*_2_) are beyond the threshold values, which implies that the growth of the population has put high pressure to the ecological situation of Baiquan County.

The calculated value of 'Status' (*B*_2_) sub-index is 0.380 in 1979, and it decreased about 40% during the period of 1979~2005 (Figure 1). In 2005, the calculated value of *B*_2_ is 0.228. The declining of 'Status' (*B*_2_) sub-index indicates that the ecological status of Baiquan County is improving from 1979 to 2005. Even so, not all the aspects of the ecological status are improving. As shown in Figure 2b, the standardized values of 'Effective Thickness of Soil Layer' (*D*_10_) increased during the years of 1979~2005. The effective thickness of black soil could affect the soil productivity directly, which has important influences on the food production and regional ecological security subsequently. It is also noticeable that the standardized values of 'Probability of Natural Disaster' (*D*_7_) for four years are beyond the threshold values, which implies great threat to the ecological status of Baiquan county.

The calculated values of 'Response' (*B*_3_) decreased from 1979 to 2000 and then increased a bit in 2005 (Figure 1). The increase of standardized values of the 'Per Capita Rural Net Income' (D_15_) and the 'Agriculture Production Per unit Cultivated Land' (*D*_16_) in 2005 may result in the increase of the calculated values of *B*_3_ in 2005 (Figure 2c). Therefore, the net income of rural people and the production of the cultivated land in Baiquan county should be improved to decrease the unsafety level of the 'Response' sub-index. It is also noticeable that the standardized values of 'Effective Irrigation Rate of Arable Land' (*D*_12_) for four years are beyond the threshold values (Figure 2c), which may imply the high vulnerability of Baiquan County to the stress of natural disasters.

### Discussion

#### Potential problems to the ecological security

Through the analysis of the assessment results, several potential problems are found to be great threat to the ecological security of Baiquan County.

Firstly, although the areas of soil and water loss control are increasing every year, the effective thickness of black soil is still decreasing. The food productivity of the parent material of the black soil is very low. The current average thickness of black soil is about 25 cm in Baiquan County. When the thickness of black soil is lower than 20 cm, the soil productivity would decrease sharply and lower the food production [16]. This would be potential risk to the food security and should be further investigated in the future.

Secondly, the application of the fertilizer is increasing with the year due to the loss of the black soil and the decrease of the productivity of the black soil in Baiquan County. The excessive application of the fertilizer has resulted in the aggravation of soil and water environmental pollution. How to adjust the fertilizer policies to reduce the application amount of the fertilizer is important to improve the environmental quality.

Thirdly, the low efficiency of the agricultural irrigation system in Baiquan County has brought high vulnerability to the draught disasters, which is frequently happened in the spring of the local areas. Therefore, the calculated values of 'Effective Irrigation Rate of Arable Land' (*D*_12_) and 'Probability of Natural Disaster' (*D*_7_) for four years are beyond the threshold values. Efficient countermeasures should be taken to improve the agricultural irrigation system in Baiquan County.

#### Effects of soil and water control on the ecological security

The soil and water control curse in Baiquan County could be divided into two stages. Before 1986, the soil and water control actions are scattered in small scales. The analysis of the historical data implies the failure of the scattered control actions during this period. For example, in the end of 1970s, the forest coverage was even under 3.7% with various natural disasters [16]. Poverty was exacerbated with the deterioration of the ecological system in that period in Baiquan County. The Baiquan county government has changed soil and water loss control polices since 1986. Comprehensive demonstration actions are taken in large scales. The area proportion of soil and water control is increased with the year. Based on the theory of ecology, comprehensive countermeasures not only bring ecological benefits but also bring economic benefits to the local areas. Thus the comprehensive soil and water loss control demonstration actions have made great contribution to improvement of the ecological status.

## Conclusions

Based on the pressure-state-response (P-S-R) framework model, the comprehensive evaluation method was proposed to investigate on the temporal variations of ecological security with Soil and Water Loss Stress in Baiquan County. The calculated values of the 'Ecological Unsafety Index' decreased from 0.701 to 0.435 during the period of 1979 to 2005. In general, the ecological security situations of Baiquan County are improving during these years. However, several problems are recognized to be the potential threats to the ecological security in the local areas. The reduction of the effective thickness of the black soil is found to be one of the potential risks to the food security of Baiquan County. The excessive application of the fertilizer has resulted in the aggravation of the soil and water environmental pollution, and how to adjust the fertilizer structure to reduce the application amount of the fertilizer is important to improve the environmental quality. The low efficiency of the agricultural irrigation system has increased the vulnerability of Baiquan County to the draught disasters, and efficient countermeasures are suggested to be taken. Through the investigation on the ecological security and its relationship with soil and water control curse, it is found that comprehensive soil and water loss control demonstration actions have made great contribution to the improvement of the ecological security.
